# PRISM: three-dimensional sub-diffractive phase-resolved imaging spectroscopic method

**DOI:** 10.1038/s41598-024-72308-z

**Published:** 2024-09-28

**Authors:** Artur Dobrowolski, Jakub Jagiełło, Beata Pyrzanowska, Karolina Piętak-Jurczak, Ewelina B. Możdżyńska, Tymoteusz Ciuk

**Affiliations:** 1 Łukasiewicz Research Network - Institute of Microelectronics and Photonics, al. Lotników 32/46, Warsaw, 02-668 Poland; 2 Faculty of Chemistry, Warsaw University of Technology, Noakowskiego 3, Warsaw, 00-664 Poland

**Keywords:** Material-phase-resolved, Imaging method, Spectroscopy, Light, Laser, Optical, Microstructures, Nanostructures, Microcolumns, Graphene, Microtrenches, Light absorption, Signal attenuation, Light scattering, Interference enhancement, Ion etched, Plasma, Atomic layer deposition, Raman spectroscopy, Scanning electron microscopy, Chemical vapor deposition, Characterization and analytical techniques, Design, synthesis and processing, Imaging techniques, Microscopy, Optical spectroscopy, Surface patterning, Graphene, Lasers, LEDs and light sources, Electronic devices

## Abstract

We demonstrate a genuine method for three-dimensional pictorial reconstructions of two-dimensional, three-dimensional, and hybrid specimens based on confocal Raman data collected in a back-scattering geometry of a 532-nm setup. The protocol, or the titular PRISM (**P**hase-**R**esolved **I**maging **S**pectroscopic **M**ethod), allows for sub-diffractive and material-resolved imaging of the object’s constituent material phases. The spacial component comes through either the signal distal attenuation ratio (direct mode) or subtle light-matter interactions, including interference enhancement and light absorption (indirect mode). The phase component is brought about by scrutinizing only selected Raman-active modes. We illustrate the PRISM approach in common real-life examples, including photolithographically structured amorphous Al_2_O_3_, reactive-ion-etched homoepitaxial SiC, and Chemical Vapor Deposition graphene transferred from copper foil onto a Si substrate and AlGaN microcolumns. The method is implementable in widespread Raman apparatus and offers a leap in the quality of materials imaging. The lateral resolution of PRISM is stage-limited by step motors to 100 nm. At the same time, the vertical accuracy is estimated at a nanometer scale due to the sensitivity of one of the applied phenomena (interference enhancement) to the physical property of the material (layer thickness).

## Introduction

Currently, we have a wide range of imaging methods. Our curiosity about the world around us drives the need to invent new methods and tools to observe what is in our surroundings. That is why we are now able to observe the universe^1^, make detailed pictures and maps of our planet, control life on it, explore the depths of oceans^2^, attend to the details of small parts, study human physique, peek into its cells, track chemical reactions, or whether we are even able to look at individual atoms^3^, or the electrons^4^ (the Nobel Prize in Physics 2023). Every telescope, camera, microscope, or detector has a construction adapted to and dictated by specific needs, phenomena, and impacts. Although the field is vast, each device is highly specialized. All that makes the current state of knowledge extremely extensive, but still, there are undiscovered areas that require the development of new tools. The Raman effect is the foundation of the Phase-Resolved Imaging Spectroscopic Method (PRISM). However, even inelastic light scattering could be useless without any additional coexisting phenomena modifying the interaction of the light with matter. In recent years, several sublime techniques have been developed to increase the measurement resolution and observe what was previously elusive. Usage of the laser in optical methods opens a wide range of possibilities. A femtosecond, or even an attosecond laser impulse, brings with it the possibility of observation of the molecule and its behaviors in an extremely short time. Nanoparticles, quantum dots, and their electron properties are measured with nonlinear effects (birefringence, Kerr effect^5^, overtones creations^4^ - Nobel Prize in Physics 2023). For accurate material imaging clearly below diffractive limits in biological samples research, a quantum photon correlation method^6^. These are some examples of using the nature of light to improve accuracy and selectivity. As with any of the above-presented methods, the PRISM method is based on an innovative idea of using the effects of light interaction with matter to introduce a new, sophisticated tool to science.

As a rule, standard optical measurements are limited by the nature of light. Object size that could be covered by optical measurement reached several hundred nanometers^7,8^. Intermediary effects methods help to get below this level^9^. In the case of the PRISM method, it is possible to reach an accuracy of single nanometers^10,11^ for thin dielectric layers or even a single-atom layer for graphene coating^12^.

Suppose we treat a light (electromagnetic wave) as a measuring medium. In that case, measuring the size of nano- and micro-structures with light is like measuring the human’s hair diameter with a ruler. It is entirely not feasible at first glance. However, light interacts with matter in many ways, and these physical phenomena allow it to break through the diffraction limits and enhance the resolution for microstructure characterization. Raman effect, interference enhancement, absorption, and distal signal attenuation are part of the phenomena above, which are fundamental mechanisms of the unconventional PRISM method. The functioning of it is illustrated through a few material phase examples, including photolithographically structured thin Al_2_O_3_, reactive-ion-etched structures in homoepitaxial SiC, and graphene on Si and AlGaN microcolumns heterostructures.

## Results

We present material-resolved imaging based on four samples with different materials and spatial structures. Each sample requires a different approach with a specialized mechanism. In the following sections, we will present examples of systems with a description of their fabrication, the mechanism used, and additional measurement methods employed.

**Fig. 1:**
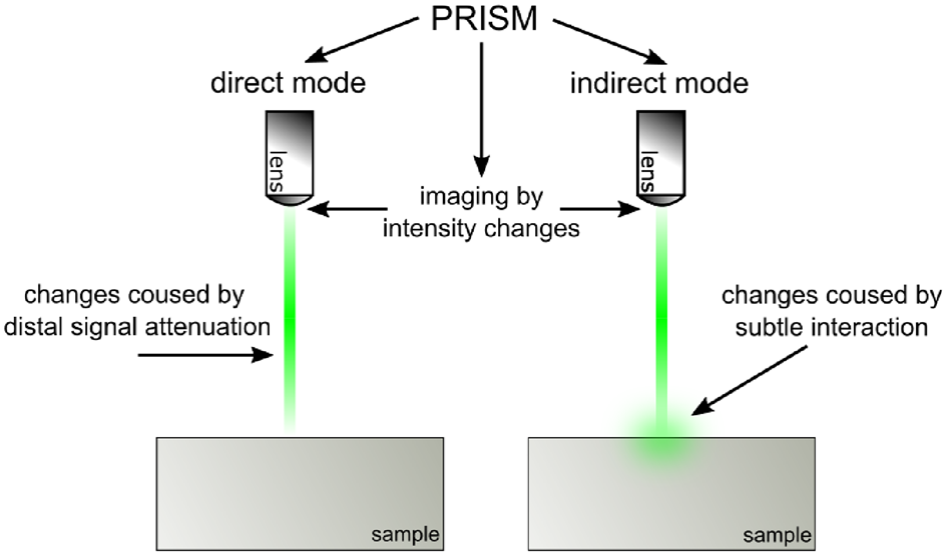
Scheme of direct and indirect modes rules.

The titular method is divided into two categories due to the complexity of the interaction of light with matter. The first category is a *direct mode*, engaging Raman signal attenuation while increasing the beam focal distance from the sample surface (Fig. 1). Determining the reference fading curve under given conditions and fitting a mathematical relationship to it is sufficient to obtain a topographic map of the surface. We will present the direct mode based on two different samples:

- a reactive-ion-etched structure in a homoepitaxial silicon carbide layer
- a graphene on aluminum gallium nitride microcolumns heterostructure

The second category is an *indirect mode*, where subtle phenomena of light interaction with matter are the foundation of the method principles (Fig. 1). We will present the second mode on the example of two samples with different structures and with different case-appropriate light phenomena:

- a thin amorphous aluminum oxide film on a bulk silicon substrate, with phenomena of interference enhancement of the Raman signal^9,12,13^
- a graphene layer transferred onto a bulk silicon substrate, with phenomena of light absorption in graphene^10,12,14^

### Direct mode

#### Reactive-ion-etched structures in a homoepitaxial SiC layer

In the case of microstructures in silicon carbide or another bulk semiconductor, surface altitude could be determined directly from the Raman signal intensity of light scattered from the sample surface. Raman signal intensity indicates the distance between the objective lens and the sample’s surface. The principle of distal attenuation of light intensity in optical systems allows a determination of that distance. The depth of field (DOF) is minimal in the confocal microscope set used in the PRISM presentation. That means the signal collected comes from one tightly constrained point, bypassing most of the signal outside the focus plane. The arrangement constructed in this way causes significant fading of the signal intensity while the sample surface moves away from the lens. The method of distal signal attenuation could be implemented for every material with Raman-active modes, and the calibration curve could be collected.

In the case of the distal Raman signal attenuation method, the necessary condition for obtaining topography from the intensity of the Raman signal is the previous preparation of a calibration curve involving a series of measurements within a certain range of the Z axis. The curve is made by finding the point (distance) of focus and gradually moving the lens away from the sample surface and the plane of focus. The curve thus formed relates the intensity (normalized to the point of full focus) of the signal to the distance of the lens from the surface. During the target mapping, the measuring table does not change its position on the Z-axis. The distance between the lens and the table remains constant throughout the measurement.

**Fig. 2:**
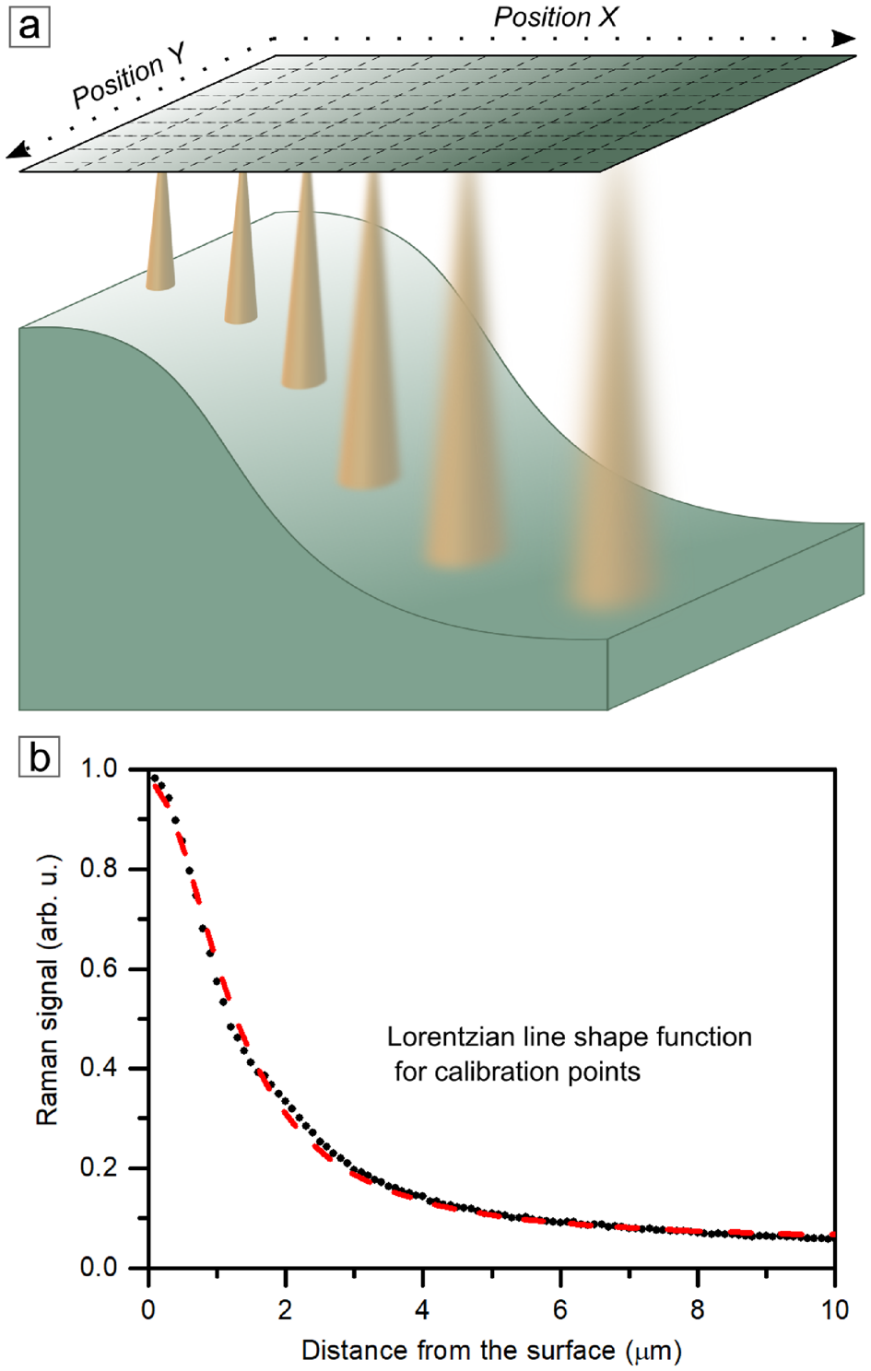
(**a**) Schematic representation of the mechanism of the PRISM method in measuring the topography of silicon carbide. The mechanism is a direct mode involving analysis of the intensity of the Raman signal scattered from the material’s surface. (**b**) The Lorentzian function from Eq. 1 fitted to reference points.

During the reference measurement, as the lens moves away from the surface, the intensity of the Raman signal is recorded (Fig. 2a). The maximal value is treated as a reference originating from the focus plane. It is possible to find a mathematical relationship that describes the behavior of the signal in the presence of a particular material. Calibration points are well-described by Lorentzian function (Fig. 2b) and given by formula (1):

1 $$\begin{aligned} y(x)=y_{0}+\frac{2A}{\pi }\frac{w}{4x^{2}+w^{2}} \end{aligned}$$

Where *x* is an argument of the function and reflects the distance between the objective lens and the material’s surface, *w* describes the distribution width, *A* is the amplitude of it, and $y_{0}$ represents the horizontal axis shift.

#### Graphene on AlGaN microcolumns heterostructure

**Fig. 3:**
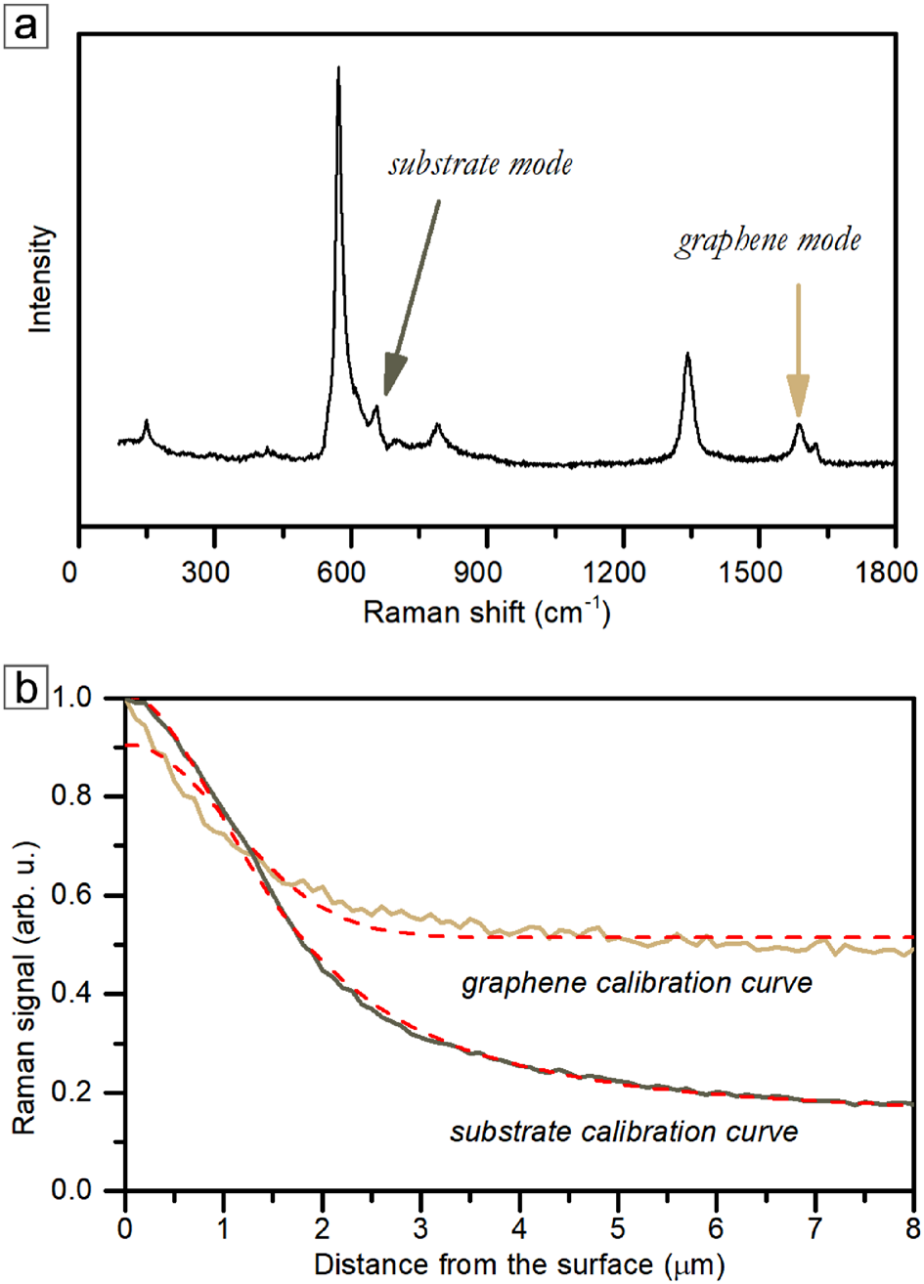
(**a**) Representative Raman spectra with graphene and the microcolumns substrate modes indicated. (**b**) Lorentzian function fitted to the reference points from the graphene on aluminum gallium nitride microcolumns heterostructure.

We used the direct mode approach with the distal signal attenuation mechanism for the graphene heterostructure presented in this section. Similar to the previous example, the calibration curve of the Raman signal fading for reference point is essential. Both heterostructure materials have their calibration curves appropriate for their optical properties (Fig. 3a and b).

### Indirect mode

#### Photolithographically-defined structures in Al_2_O_3_

**Fig. 4:**
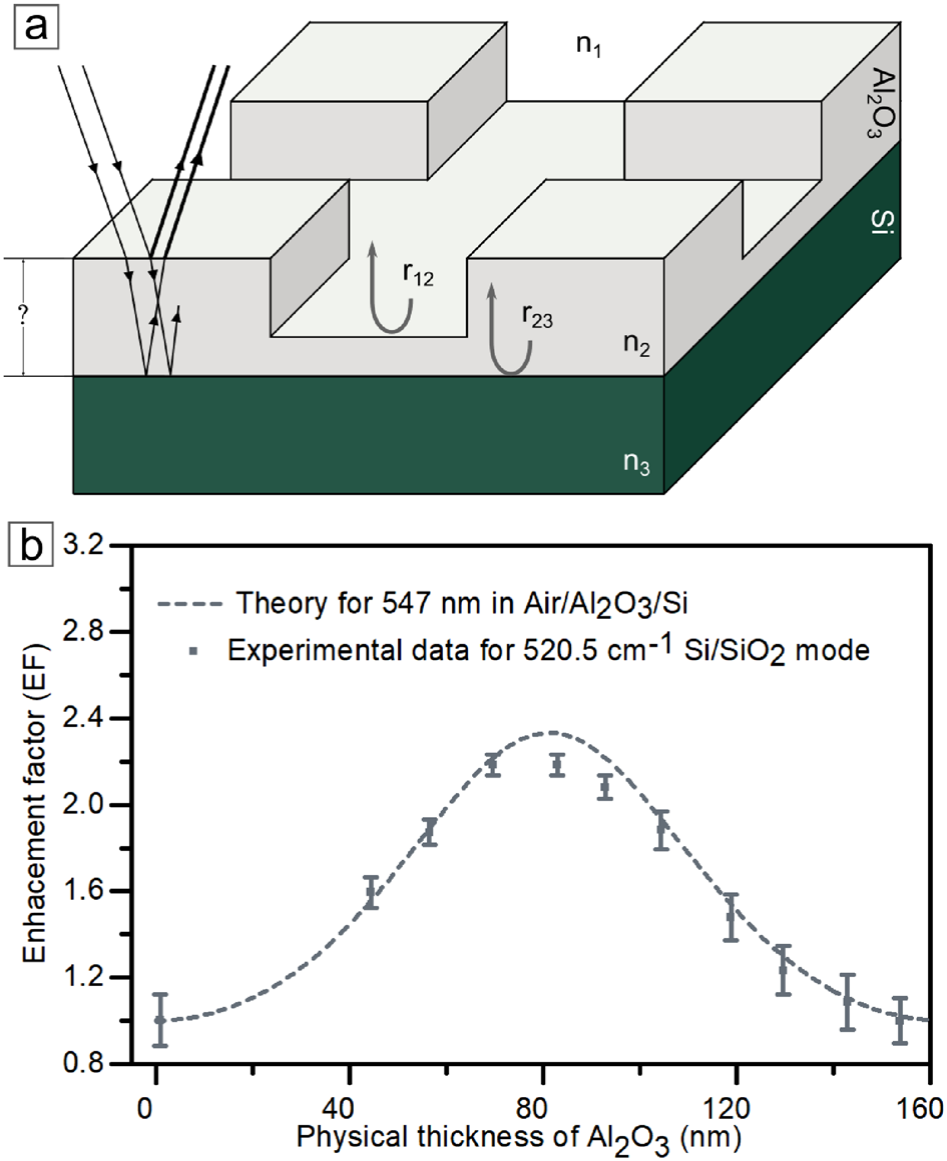
(**a**) Simplified scheme of the indirect interaction method. Interference enhancement of the Raman signal is a fundamental phenomenon for topographic imaging of the thin aluminum oxide layer. (**b**) Calibration curve of Raman signal enhancement based on the formula from equation (Eq. 2).

Interference enhancement of the Raman signal^9,11,13^ plays an essential role in the functioning of the PRISM method for thin aluminum oxide films. The phenomenon occurs in a thin dielectric layer, where layers interface, varied distribution of refractive indices, and a precisely defined difference in optical paths predispose a backscattered light to constructive interference and enhancement (Fig. 4a). We introduce the enhancement factor $EF(x)= \frac{I(x)}{I_{ref}}$ to formalize that effect. This coefficient reflects the ratio of light intensities returning after scattering to the referenced light signal from solely the substrate (without the top Al_2_O_3_ layer). Identification of signal origin involves the Raman effect, whose spectra are a kind of fingerprint for each material. The enhancement factor correlates with layer thickness, as described in the formula below (Eq. (2))^9,11^:

2 $$\begin{aligned} \begin{aligned} EF(v_{e},v_{s},x)= \bigg | \frac{[1+r_{12}(v_{e})r_{23}(v_{e})]}{[1+r_{12}(v_{e})r_{23}(v_{e})e^{-2i\delta (v_{e},x)}]}*\\ *\frac{[1+r_{12}(v_{s})r_{23}(v_{s})]}{[1+r_{12}(v_{s})r_{23}(v_{s})e^{-2i\delta (v_{s},x)}]} \bigg |^2 \end{aligned} \end{aligned}$$

The symbol $r_{ij}$ in Eq. (2) is a reflection coefficient for normal incidence (backscattering mode) described by $r_{ij}(v)= \frac{n_{i}(v)-n_{j}(v)}{n_{i}(v)+n_{j}(v)}$, where $n_{i,j}$ is a refractive index of each material with dispersion relation for incident light ${532}\,\hbox {nm}$ ($v_{e}$- **e**xcitation) and scattered light ($v_{s}$- **s**cattered) for ${547}\,\hbox {nm}$ wavelength of silicon substrate Raman active mode ($\omega _{Si}={520.5}\,\hbox {cm}^{-1}$). Furthermore, $\delta (v,x)=2\pi n_{2}(v)xv$, where *x* is the thickness of the layer, *v* is a spectroscopic wave number, and the whole is a factor related to the spatiality of the system.

The obtained function is a kind of theoretical reference curve (Fig. 4b)). While experimenting, we are following the intensity of a Raman-active mode from the substrate under the thin Al_2_O_3_. Then, it is compared with the reference signal of the substrate material without the Al_2_O_3_ layer presence. Finally, the ratio of these two values, set as an Enhancement Factor, allowed us to determine (Eq. 2) the layer thickness $EF(x) \rightarrow ~x$.

#### Transferred graphene on SiO_2_/Si

**Fig. 5:**
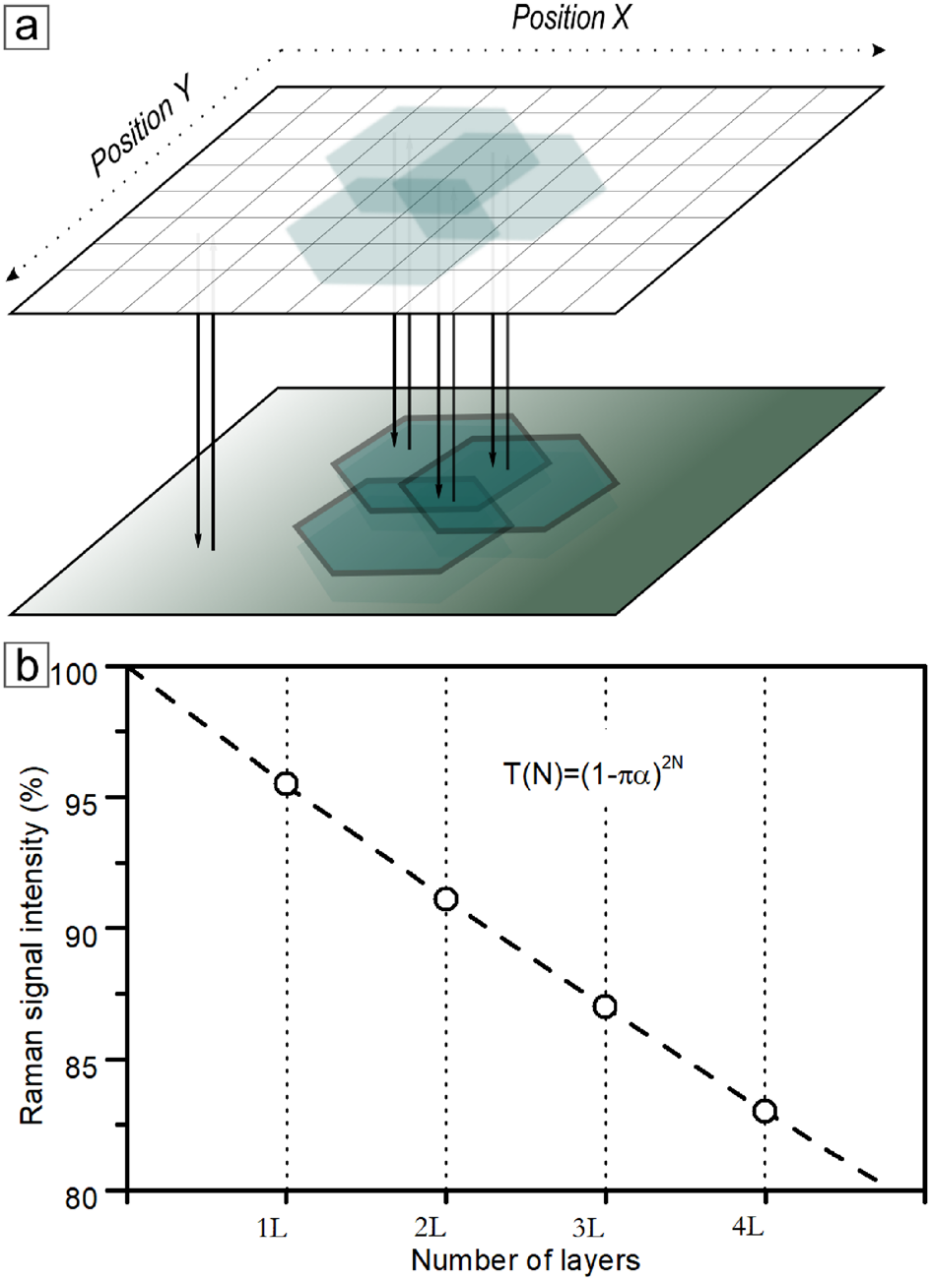
(**a**) Simplified scheme of the indirect method based on light absorption in graphene. (**b**) Calibration curve of the Raman signal intensity as a function of the number of the graphene layers.

Light passing through the atomically thin layers attenuated its intensity. The absorbed portion of the light is strictly connected with the fine-structure constant and is equal to $\pi \alpha \approx {2.3}{\%}$. In the optical system with a backscatter geometry, light passes through the graphene layers twice each time, losing respectively more of the intensity (Fig. 5a)^12,15^. Taking into account the issue of the fine structure constant and backscattering, it leads to a formula linking the intensity of the Raman signal of the substrate on which the graphene rests with the number of its layers (Fig. 5b):

3 $$\begin{aligned} T(N)=(1-\pi \alpha )^{2N} \end{aligned}$$

Where N is the number of graphene layers and $\alpha$ is a fine-structure constant.

## Discussion

### Direct mode

**Fig. 6:**
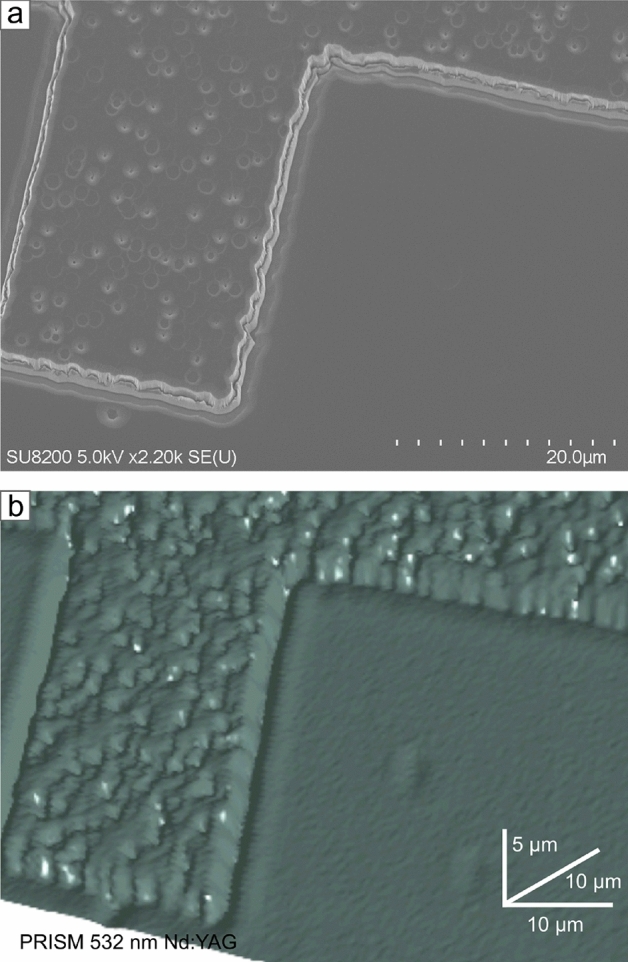
(**a**) Scanning electron microscopy with secondary electrons detector image of a reactive-ion-etched structure in homoepitaxial silicon carbide. (**b**) 3D imaging effect of the PRISM method based on the distal Raman signal attenuation on the homoepitaxial microstructure.

The example of the silicon carbide microstructures perfectly demonstrates the PRISM method’s accuracy and the possibilities it offers. Beyond the apparent aspect of a visual capture of the structure on a micro-scale, there are also structural defects of the material highlighted (Fig. 6b). We have detected surface defects related to the epitaxial process (threading screw dislocations, threading edge dislocation TED)^16–19^. Spatial imaging allows tracking changes in point defects until the etching process. Even at shallow etching depths, defects become visible, and it is possible to determine the origin of their formation. In structures obtained with the ICP-RIE method, it is more critical to track defects of the etching process, which is erosion in the shape of ducts and cavities at the edge of the etched area, known as microtrenches^20,21^. The shape and nature of micro trenches depend on etching process conditions, especially the plasma parameters. The forming of microtrenches is an unwanted effect affecting the electric field and consequently worsening the electrical properties and shortening the life of the device. A definite advantage of PRISM imaging over an optical or electron microscopy image (Fig. 6a) is that in a single measurement, we obtain spatial data from which a three-dimensional model is built.

**Fig. 7:**
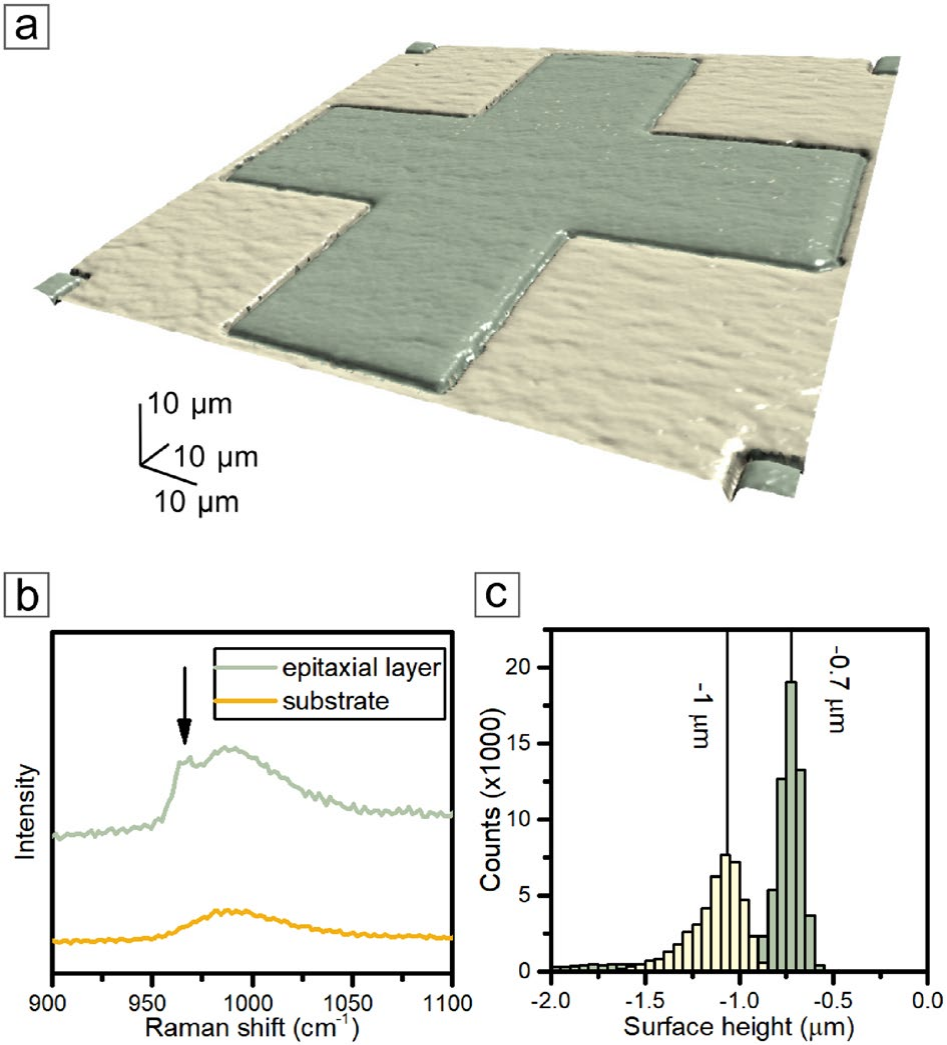
(**a**) Material-resolved PRISM imaging of the structure of a homoepitaxial layer that differs from the substrate in the level of free charge carriers concentration. (**b**) Spectral differences that allow separation of layers with different levels of concentration of free charge carriers. (**c**) Separation of the dataset for both layers of the structure.

If the etching process was long enough to etch through the epitaxial layer to the substrate layer, material-resolved imaging of both layers could be performed simultaneously. Although the two materials are identical in terms of crystal structure (4H-SiC homoepitaxial layer) and should not show differences in Raman spectra, the difference in the concentration level of free charge carriers affects the efficiency of coupling one of the optical modes with the plasmons^22^ and, as a result, the shape and position of the coupled mode (Fig. 7b), which is a criterion for the identification and separation of layers (Fig. 3.1a). Assigning a label based on spectral differences makes it possible to separate the data set and accurately determine the average heights of the two layers (Fig. 7c).

**Fig. 8:**
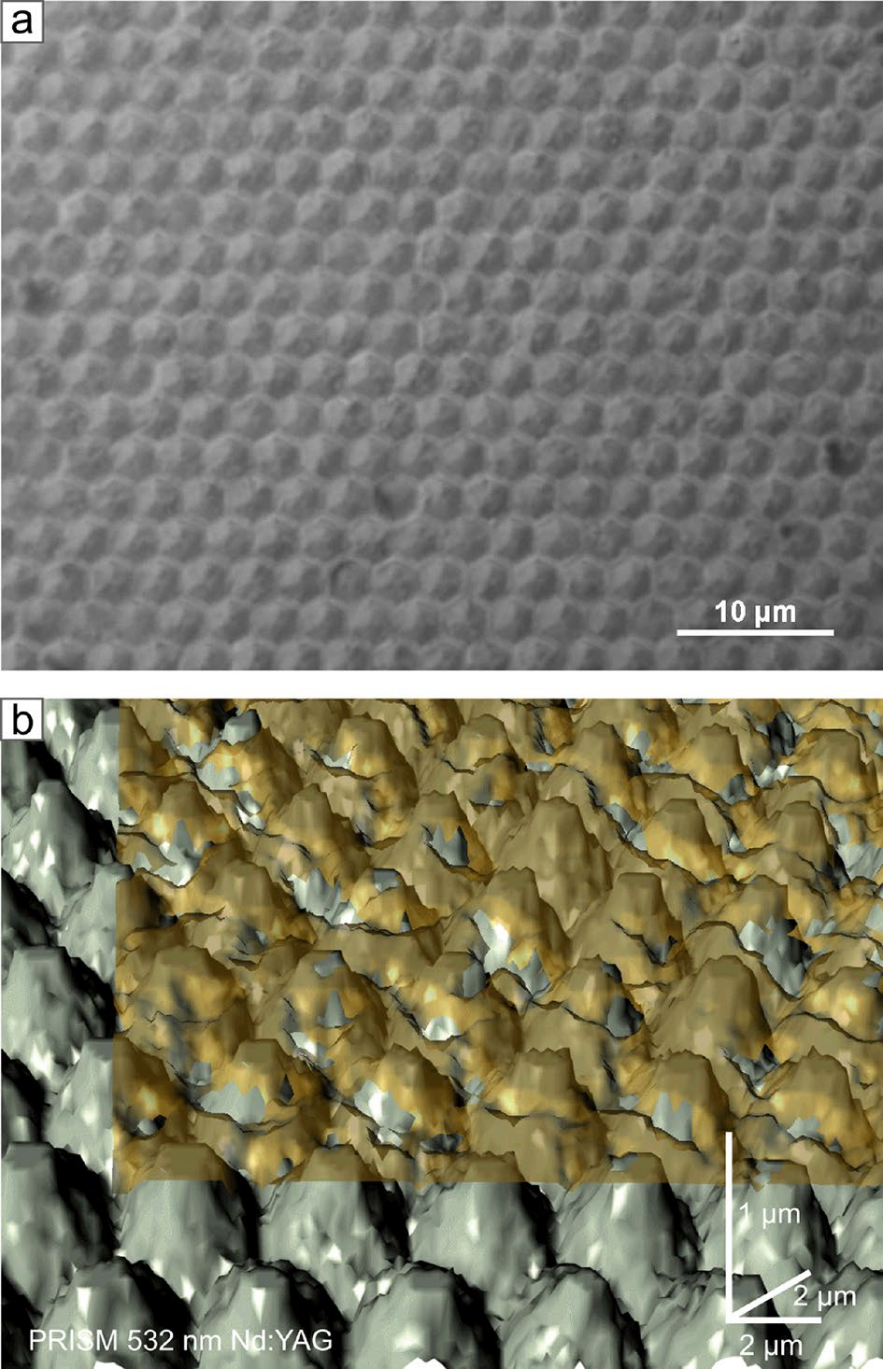
(**a**) Optical microscope with bright field mode and differential interference contrast of the graphene on aluminum gallium nitride microcolumns heterostructure. (**b**) Corresponding imaging results of the PRISM method with distal Raman signal attenuation mode with material components separation possibility for the graphene heterostructure.

Graphene heterostructures find many applications in LED technologies, photovoltaic, and technological sensors^23,24^. The presented example aimed, in particular, at the use of optoelectronics. The case of a graphene sheet covering the spatial structure of microcolumns is fascinating regarding the complexities of surface topography and interdependent material attachment to each other. The graphene sheet hangs up on top of the microcolumns (Fig. 8a), and its surface is not completely flat. It contains cavities related to free hanging upon free space between columns. Thanks to material-resolved imaging, it is possible to separate each layer visually (Fig. 8b). That material-resolved analysis is especially useful when the point of contact is key. The atomically thin carbon layer in that heterostructure is a kind of electric jumper for individual, isolated columns. That is why properly adhering graphene to the microcolumns is so important. Separating the images of the individual layers makes verifying the correct alignment of the materials easier.

### Indirect mode

**Fig. 9:**
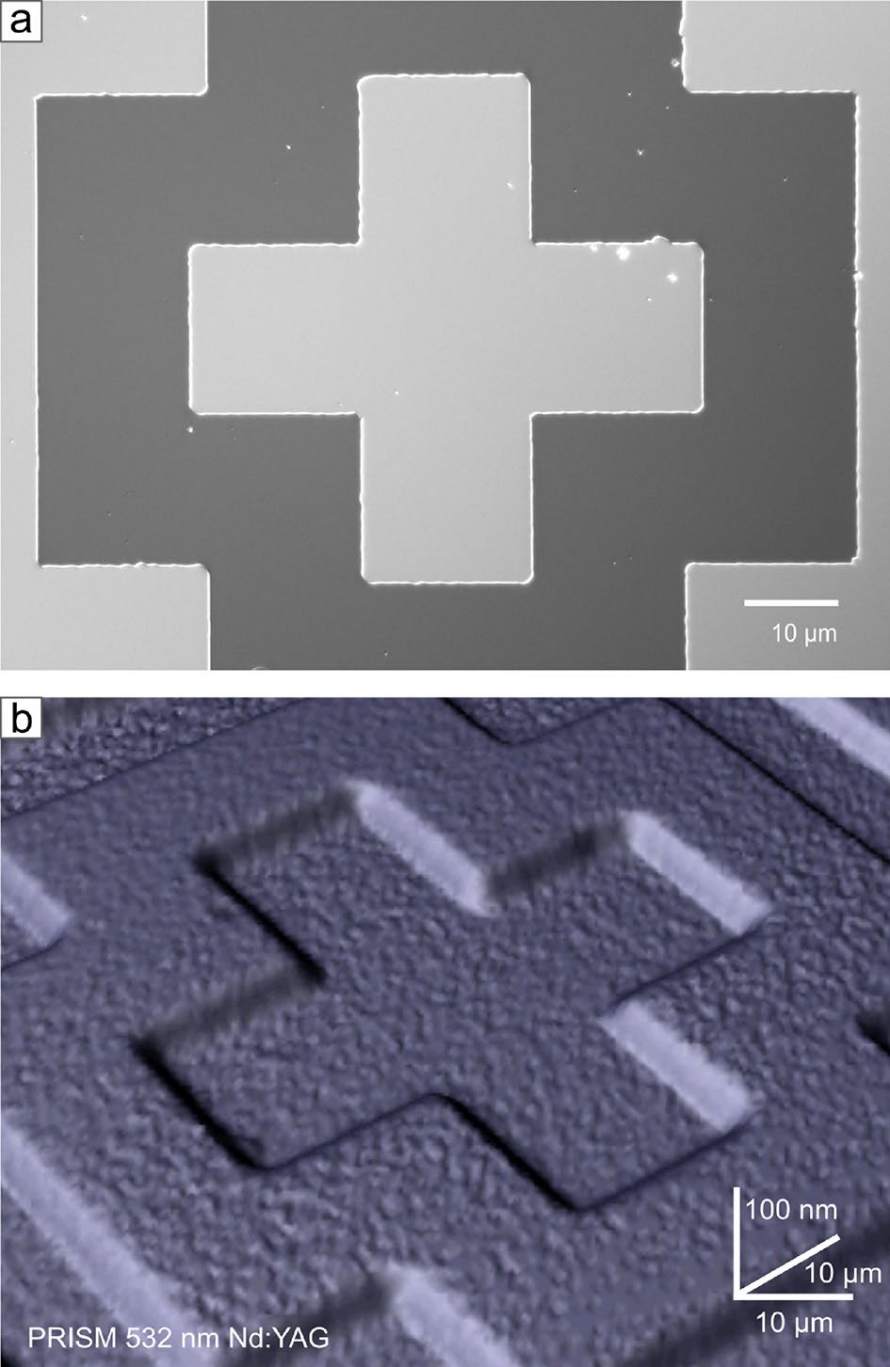
(**a**) Optical microscope image with bright field mode and differential interference contrast of the structure in a thin aluminum oxide layer. (**b**) Results from the PRISM imaging with an indirect method based on the interference enhancement of a Raman signal for microstructures in a thin aluminum oxide layer.

Conversion of the intensity in a given map point to the layer thickness leads to the spatial model of the layer. The test sample for thin Al_2_O_3_ in the indirect model contains a framed, concave cross (Fig. 9b). PRISM model shows a high-accuracy convergence with a standard optical image (Fig. 9a). Although both images correspond strictly to each other, the optical image is limited to the view from one perspective, which can only suggest shapes without any spacious information.

Two factors limit horizontal resolution (stage movement in horizontal axes). The first limitation originates from the stage movement and mechanical limits reaching ${0.1}\,{\mu \hbox {m}}$. The second restriction involves the laser spot diameter projected on the sample in the focus plane and reaching a few hundred nanometers. Estimating resolution in the vertical axis (now referred to as the Z axis) is indirectly related to the detector sensitivity. Since the layer thickness is calculated based on a scattered light signal intensity, the measurement accuracy links mainly to the CCD camera sensitivity in signal analysis.

**Fig. 10:**
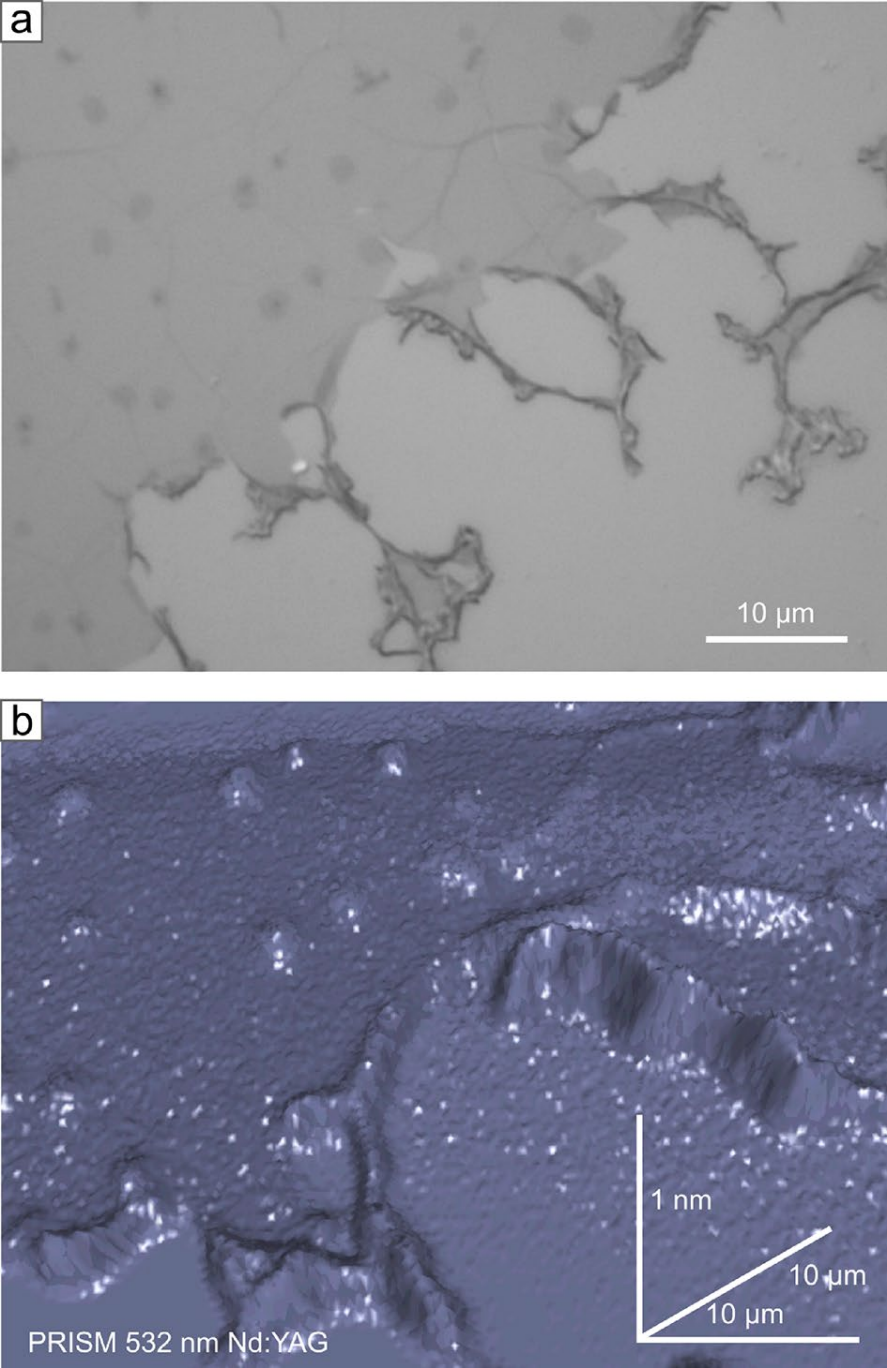
(**a**) Optical microscope with bright field mode and differential interference contrast of the transferred graphene layer onto silicon bulk substrate. (**b**) Imaging results of the PRISM method with the effect of light absorption of graphene.

It is uncommon to be able to observe and discover details of atomically thin layers with an optical instrument. With that perspective in mind, the diffraction limits were overcome, and the imaging resolution was significantly improved. Indeed, with the indirect mode and additional unique properties of graphene, it is possible to determine the number of graphene layers within the visible-light range. Imaging from the Fig. 10 demonstrates the resolution capabilities of the method. The method is highly sensitive in terms of analysis of topographical defects like fraying, overlapping, and additional layers (Fig. 10b). Having the graphene-free region as a reference, we can precisely determine the number of layers for a single graphene transfer.

**Fig. 11:**
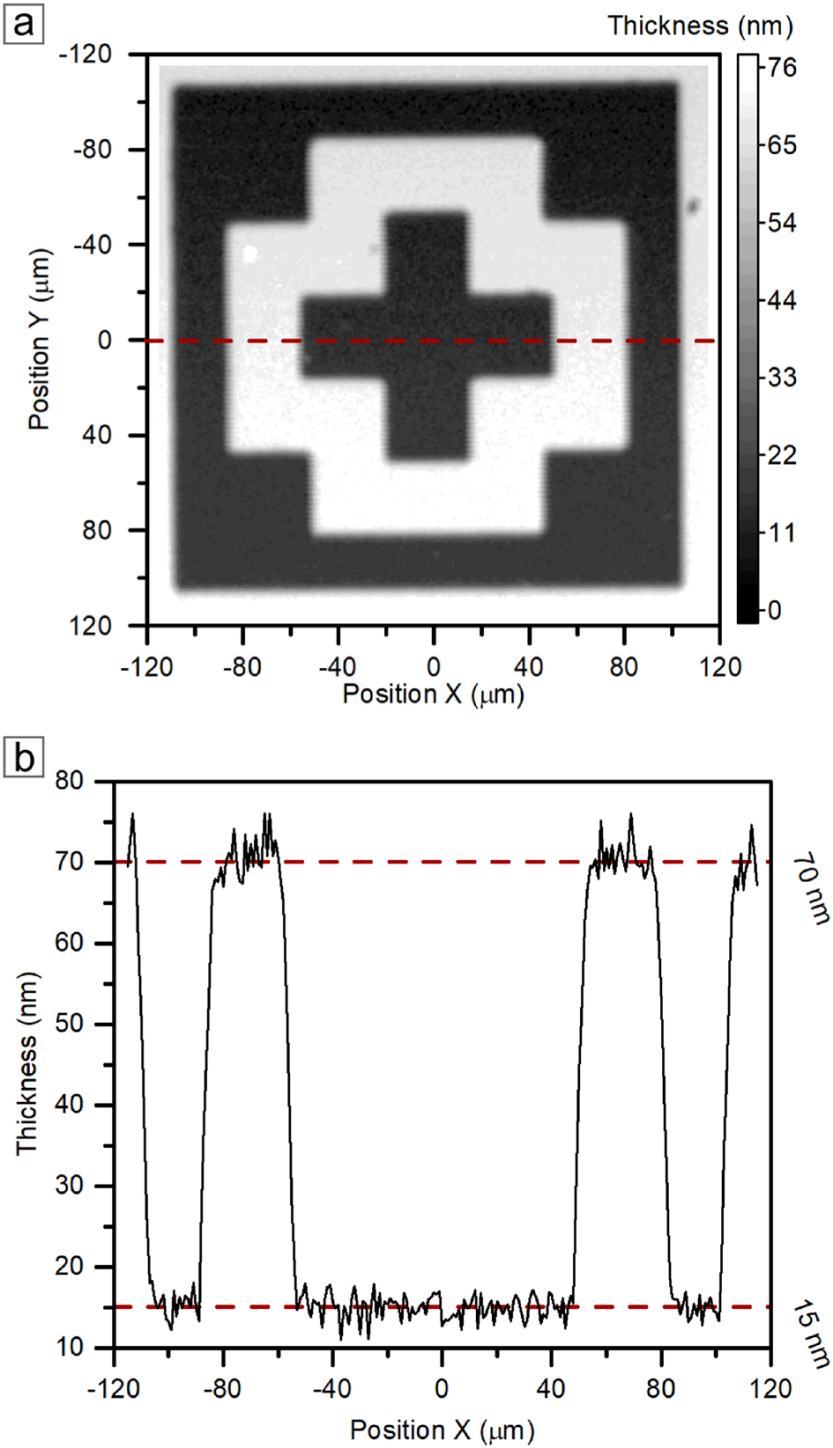
(**a**) Results of the PRISM imaging as a 2D contour map of the grey scale of the aluminum oxide layer thickness with marked height profile (dashed line). (**b**) Cross-sectional height profile obtained from the PRISM imaging.

Data collected for the 3D imaging of photolithography structures in a thin Al_2_O_3_ could also be essential in deeper analysis and precise designation of layer thickness or altitude of structure surface. In Fig. 11a, we are demonstrating the results of imaging as a flat, two-dimensional contour map to show the area of sampling for profile (Fig. 11b). Information presented on the profile unambiguously indicates that the Al_2_O_3_ layer of the test sample is ${70}\,\hbox {nm}$ on the top of the structure and ${15}\,\hbox {nm}$ in the cavities. Therefore, the difference between the maximal and minimal values is ${55}\,\hbox {nm}$.

Optical instruments have notable restrictions in resolution due to the diffraction limits. That limitation defines resolution on the order of several hundred nanometers, and the precision of imaging of nano- and micro-structures is insufficient. An appropriate approach could help break through the limitation and gain an optical method with almost atomic resolution. In this work, we have presented a phase-resolved, three-dimensional topographic imaging method that overcomes diffraction limitations. The presented method is a twofold system with direct and indirect modes. In direct mode, the distal Raman signal attenuation is a primary mechanism for topographic imaging. That approach is available for every solid-state material with Raman-active mode. Vertical resolution is related to the sensitivity of the camera detector in Raman microscopy. We made a 3D imaging of etched microstructures in SiC and graphene heterostructure on microcolumns array to demonstrate how the direct mode works. We observed a few micrometers of structural defects in the first sample and the detailed topography of an alignment of graphene on the microcolumns in the second sample. The indirect mode of the PRISM method uses additional effects of light interaction with matter. As a result of applying this mode in imaging a thin Al_2_O_3_ layer, we obtain a resolution in the order of several nanometers. When we used an absorption attenuation of a Raman signal in graphene, we observed details in the atomically thin layer.

The multitude of physical phenomena occurring during optical measurement urges particular caution. Although the method is based on variety of phenomena, one should be aware that not all of them can be transformed into a functional element and remains a parallel parasitic effect that disturbs the perception of the resulting image. On the impact that emerged during the development of the manuscript should be mentioned:

- Phonon-plasmon coupling in silicon carbide. In polar semiconductors, the phonon from the optical branch (here *LO* mode near ${964}\,{\hbox {cm}^{-1}}$) effectively couples with the free-carrier plasmons causing a change in the mode shape, broadening it and shifting it toward higher energies as the free-carrier concentration increases. Aware of the problem, we focused on imaging based on modes from a different range, which are less susceptible to the physical properties of the material and its interaction with light. The *LO* mode (or more precisely, its coupled version *LOPC*) served us as an indicator of layers with different concentrations of free charge carriers for phase separation^22^.
- Shape variation of Raman modes due to the composition ratio of AlGaN composition. In structures with varying compositional ratios of equivalent elements, changes in the Raman spectrum can be expected, if only due to a change in the crystal vibrational structure. In the case of the $Al_xGa_{1-x}N$ structure, the mode that changes its character in a stepwise manner is the $E_2$ mode near ${600}\,{\hbox {cm}^{-1}}$. To avoid the problem, a common for the whole structure $A_1$ mode near ${850}\,{\hbox {cm}^{-1}}$ was chosen^25^. As in the previous example, appropriate analysis can enable the separation of phases with different compositions.
- Effects of strain and free charge carriers concentration on graphene modes. The effects of free-carrier concentration and stress on the correlation of G and 2D graphene modes positions have been precisely determined for transferred graphene on SiO_2_/Si substrates^26^. The concept has also been successfully extended to other types of graphene. Even if a correlation of 2D modes intensity depending on the local physical properties of the material can be found, the effect on the image is so marginal that it has not been isolated in this work.
- Local electric field in the grain boundary and sharp edges. While preparing images for etched structures in homoepitaxial SiC layers, a new peculiar mode was noticed at the boundary and edge areas. However, this element is a small part of the image, so it was omitted without affecting the overall impression of the image.

To sum up, gaining accuracy below the visible light range diffraction limits is achieved in this work by the following approach:

- In the thin amorphous Al_2_O_3_ layer sample, we obtain a detailed image of a layer thinner far below ${100}\,\hbox {nm}$ by the interference enhancement of incident and scattered light. It is not feasible with a standard microscope, for instance, by imaging the cross-section of a structure. The details are so small that they are invisible under the standard microscope.
- In the sample of transferred graphene, Raman signal intensity changes caused by light absorption could be noticeable even with the presence of just a single layer (atomically thin changes).

## Methods

### Reactive-ion-etched structures in a homoepitaxial SiC layer

#### Sample preparation

Creating silicon carbide microstructures involves the Top-Down method, which is non-trivial in this situation. Silicon carbide is highly resistant to standard chemical etching forms due to its chemical properties. Etching with reactive ions with inductively coupled plasma (ICP-RIE) is a dry etching technique suitable for this highly resistant material. For this PRISM method mode, we have prepared microstructures in a homoepitaxial layer of SiC in the form of an isosceles cross (dimensions of ${20}\,{\mu \hbox {m}}\times {90}\,{\mu \hbox {m}}$) etched with reactive ions.

The nitrogen-doped 5.34-$\mu$m-thick homoepitaxial layer was grown on a ${15}\,\hbox {mm}\times {15}\,\hbox {mm}$ sample cut from a 3-in conducting, n-type (4$\times$10^18^ cm^-3^), 364-$\mu$m-thick 4H-SiC substrate (SiCrystal GmbH) with a ${4}\,^{\circ }$ off-cut from the basal vector [0001] towards the [11-20] direction. Silane and propane were used as precursors (*C* : *Si* = 1.8), and hydrogen as carrier gas^27^. Before the growth, the SiC surface was in-situ etched with a hydrogen and propane mixture^28^. The process was carried out in a hot-wall Aixtron VP508 reactor, and the epitaxial layer net donor concentration $N_D - N_A$, where $N_D$ is the bulk donor concentration, and $N_A$ is the bulk acceptor concentration, estimated at 6.9$\times$10^15^ cm^-3^ with the voltage-capacitance method. Then, using UV400 optical lithography, cross-shaped patterns were defined on top of the epitaxial layer, and a 200-nm-thick Cr etching mask electron-beam-evaporated. The thin Cr layer served as a mask in the ICP-RIE process.

#### Measurement details

Renishaw’s inVia confocal Raman microscope system serves, for each example included in the content, to obtain the Raman spectrum, the basis of the PRISM method.

For 3D imaging of the microstructures in SiC, we used a ${100}\times$ objective with a numerical aperture of $NA=0.85$, ${532}\,\hbox {nm}$ Nd:YAG laser with ${100}{\%}$ power of ${13.5}\,\hbox {mW}$ cast on the sample’s surface during an exposure of $1\times {0.5}\,\hbox {s}$. We have collected data from ${96}\,{\mu \hbox {m}}\times {96}\,{\mu \hbox {m}}$ area with ${0.3}\,{\mu \hbox {m}}$ step. That generated 103041 measuring points during a measurement lasting 14.3 h.

The 3D image was built on the intensity of the *FTO* mode around ${777}\,{\hbox {cm}^{-1}}$. As a different phase indicator (substrate and homoepitaxial layer), the *LOPC* mode near ${964}\,{\hbox {cm}^{-1}}$ was used^22^.

We compared the scanning electron microscope (SEM) image with the PRISM results. Electron waves are essential to illustrate the detail of the structure and to compare with the accuracy that the PRISM method offers. Using the secondary electrons detector (SE(U)), acceleration voltage in the range of 2.0 to ${5.0}\,\hbox {kV}$, and stage tilt to make an angle view, we have obtained a high-resolution image with details that could not appear in a standard planar view.

### Graphene on AlGaN microcolumns heterostructure

#### Sample preparation

We made the graphene heterostructure using a two-stage process. First was the growth of an evenly distributed, uniform array of the AlGaN microcolumns on a pre-patterned c-plane sapphire substrate (PSS) under the low-pressure epitaxial process of metalorganic deposition (LP-MOVPE)^29^. The second stage was to obtain a continuous graphene layer with the conventional method of Chemical Vapor Deposition (CVD) on copper foil^30^, followed by the polydimethylsiloxane (PDMS) frame-assisted transfer.

#### Measurement details

Raman measurement in the presence of graphene needs special attention due to the difficulties in obtaining a good-quality spectrum. The relative closeness of Raman-active modes allows the collection of signals of the graphene layer and underneath AlGaN microcolumns materials at one measurement.

The imaging was supported by two modes, AlGaN $A_1$ mode near ${850}\,{\hbox {cm}^{-1}}$ and graphene G mode around ${1580}\,{\hbox {cm}^{-1}}$.

The measurement conditions prepared for graphene measurement will also be enough for AlGaN. We used an objective lens of ${100}\times$ magnitude with ${100}{\%}$ power of ${532}\,\hbox {nm}$ Nd:YAG laser with an exposition of $1\times {1.5}\,\hbox {s}$. We have collected data from a ${24}\,{\mu \hbox {m}}\times {24}\,{\mu \hbox {m}}$ area with ${0.15}\,{\mu \hbox {m}}$, which generated 25921 measuring points, during 11 h of measurement.

Nikon LV150N Differential Interference Contrast (DIC) optical microscopy in Bright Field (BF) under ${100}\times$ magnification objective images supplements the PRISM method.

### Photolithographically-defined structures in thin amorphous Al_2_O_3_

#### Sample preparation

We used the atomic layer deposition method (ALD), realized in a 4-in Picosun R200 reactor at ${300}\,^\circ \hbox {C}$, to synthesize a thin layer of amorphous aluminum oxide. Before producing the target Al_2_O_3_ layer, the stage was to prepare the silicon substrate properly by removing the pristine silicon oxide layer with a hydrofluoric acid rinsing. Then, we used trimethylaluminum (TMA) and de-ionized water (DI) as aluminum and oxygen precursors, respectively. The number of process cycles can strictly correlate with the obtained layer thickness (in this case, it is several dozen nanometers). Finally, we prepared the target patterns in layers with positive photolithography. To present the method, we have prepared a test structure based on a photolithographic template of alignment marks in the form of framed concave crosses with shoulder dimensions of ${40}\,{\mu \hbox {m}}\times {120}\,{\mu \hbox {m}}$ (${220}\,{\mu \hbox {m}}\times {220}\,{\mu \hbox {m}}$ external dimension of the frame, also concave).

#### Measurement details

Si-substrate-related mode at ${520}\,\hbox {cm}^{-1}$ indicates Raman signal enhancement on thin Al_2_O_3_ layers.

Getting good quality data for 3D analysis requires strictly defined measurement conditions. A particular focus should be on the imaging area and resolution for visual quality. We have made a mapping with a resolution of ${1}\,{\mu \hbox {m}}$ on the squared area of ${230}\,{\mu \hbox {m}}\times {230}\,{\mu \hbox {m}}$, giving 53,361 measurement points. A more critical role for method implementation is the exposure conditions and all settings related to the optical system. At first, Nd:YAG ${532}\,\hbox {nm}$ wavelength laser with ${100}{\%}$ of ${13.5}\,\hbox {mW}$ input power enables the observation of the Raman effect. ${20}{\times }$ magnification objective with NA=0.40 numerical aperture provides proper projections and collections of the light signal. Exposure of 2 acquisition of ${0.1}\,\hbox {s}$ measurement was responsible for a sufficiently strong and clear Raman signal. These measurement settings allow one to obtain the presented image within 3 h.

Nikon LV150N Differential Interference Contrast (DIC) optical microscopy in Bright Field (BF) under ${50}{\times }$ magnification objective images supports PRISM results to highlight the detail of the presented method.

### Transferred graphene on SiO_2_/Si

#### Sample preparation

Graphene transfer from a copper substrate onto a target substrate is an accepted protocol for obtaining graphene samples. The material imaged here is graphene grown by Chemical Vapor Deposition (CVD) on a metallic substrate - copper foil (Graphenea). The graphene was spin-coated with anisole solution (4 %) of poly(methyl methacrylate) (PMMA), detached from copper through high-speed electrochemical delamination in 1.0-M KCl solution, transferred onto the target substrate, here $SiO_{2}/Si$, and rinsed off the PMMA residue with warm acetone^30^.

#### Measurement details

While the layer thickness determination mechanism is based on the substrate Raman-active signal attenuation by the atomic layer absorption, we need a back-scattered geometry optical system. That is also a significant advantage in the case of thick, bulk, and opaque substrate materials. Back-scatter geometry allows measurement without complicated and destructive sample preparation. We consider the *Si* Raman mode near ${520}\,\hbox {cm}^{-1}$ for graphene transferred into the silicon substrate. Its signal is strong, which helps to compensate for a measurement time. Exposition of $1\times {0.1}\,\hbox {s}$ with ${532}\,\hbox {nm}$ Nd:YAG laser and ${100}{\%}$ of ${13.5}\,\hbox {mW}$ power cast by ${100}{\times }$ objective with a numerical aperture of $NA=0.85$ is enough to collect good-quality information. We have made a mapping with a resolution of ${0.15}\,{\mu \hbox {m}}$ on the area of ${48}\,{\mu \hbox {m}}\times {32}\,{\mu \hbox {m}}$, giving 68,694 measurement points collected in a measurement lasting 2 h. Differential Interference Contrast (DIC) optical microscopy in Bright Field (BF) under 100x magnification objective images supports the PRISM method.

## Data Availability

The data that support the findings of this study are available from the corresponding author upon reasonable request.
